# High-Throughput Sequencing Reveals H_2_O_2_ Stress-Associated MicroRNAs and a Potential Regulatory Network in *Brachypodium distachyon* Seedlings

**DOI:** 10.3389/fpls.2016.01567

**Published:** 2016-10-20

**Authors:** Dong-Wen Lv, Shoumin Zhen, Geng-Rui Zhu, Yan-Wei Bian, Guan-Xing Chen, Cai-Xia Han, Zi-Tong Yu, Yue-Ming Yan

**Affiliations:** ^1^College of Life Science, Capital Normal UniversityBeijing, China; ^2^Department of Oral and Craniofacial Molecular Biology, VCU Philips Institute for Oral Health Research, School of Dentistry, Virginia Commonwealth UniversityRichmond, VA, USA; ^3^State Agriculture Biotechnology Centre, Murdoch UniversityPerth, WA, Australia

**Keywords:** Bd21, H_2_O_2_ stress, microRNA, high-throughput sequencing, regulatory network

## Abstract

Oxidative stress in plants can be triggered by many environmental stress factors, such as drought and salinity. *Brachypodium distachyon* is a model organism for the study of biofuel plants and crops, such as wheat. Although recent studies have found many oxidative stress response-related proteins, the mechanism of microRNA (miRNA)-mediated oxidative stress response is still unclear. Using next generation high-throughput sequencing technology, the small RNAs were sequenced from the model plant *B. distachyon* 21 (Bd21) under H_2_O_2_ stress and normal growth conditions. In total, 144 known *B. distachyon* miRNAs and 221 potential new miRNAs were identified. Further analysis of potential new miRNAs suggested that 36 could be clustered into known miRNA families, while the remaining 185 were identified as *B. distachyon*-specific new miRNAs. Differential analysis of miRNAs from the normal and H_2_O_2_ stress libraries identified 31 known and 30 new H_2_O_2_ stress responsive miRNAs. The expression patterns of seven representative miRNAs were verified by reverse transcription quantitative polymerase chain reaction (RT-qPCR) analysis, which produced results consistent with those of the deep sequencing method. Moreover, we also performed RT-qPCR analysis to verify the expression levels of 13 target genes and the cleavage site of 5 target genes by known or novel miRNAs were validated experimentally by 5′ RACE. Additionally, a miRNA-mediated gene regulatory network for H_2_O_2_ stress response was constructed. Our study identifies a set of H_2_O_2_-responsive miRNAs and their target genes and reveals the mechanism of oxidative stress response and defense at the post-transcriptional regulatory level.

## Background

Many environmental stress factors, including high light, UV irradiation, heat, salinity, drought, and cold, can cause plant cells to produce reactive oxygen species (ROS), leading to acceleration of lipid peroxidation and leaf senescence (Mittler et al., 2004; Upadhyaya et al., 2007). Hydrogen peroxide (H_2_O_2_) is a kind of ROS mainly produced by the following parts of the cell: the mitochondrion, peroxisome, chloroplast, apoplast, and plasma membrane (Apel and Hirt, 2004). Superoxide radicals (${\text{O}}_{2}^{-}$), another kind of ROS, can also be rapidly dismutated to H_2_O_2_ spontaneously or catalyzed by superoxide dismutase (SOD). In contrast to other ROS, such as ${\text{O}}_{2}^{-}$ and hydroxyl radicals (OH^−^), H_2_O_2_ can easily pass through membranes (Foyer et al., 1997; Uchida et al., 2002; de Azevedo Neto et al., 2005; Wahid et al., 2007) and is relatively stable, so it is suitable for its roles as an important component of cell signaling cascades (Mittler, 2002; Neill S. et al., 2002; Neill S. J. et al., 2002; Vranová et al., 2002) and an indispensable second messenger in biotic and abiotic stress responses (Pastori and Foyer, 2002). H_2_O_2_ stress can affect fluctuation of the Ca^2+^ concentration in plants, thereby inducing the production of appropriate amounts of antioxidants (Rentel and Knight, 2004). Global transcript profiling under H_2_O_2_ stress in tobacco revealed that redox homeostasis associated proteins are upregulated while proteins related to normal growth and development are downregulated (Vandenabeele et al., 2003). H_2_O_2_ can also trigger acclimation and cross-tolerance phenomena (Neill S. et al., 2002; Pastori and Foyer, 2002). Overall, at low concentrations H_2_O_2_ may play a role as a signaling molecule, whereas at high concentrations H_2_O_2_ will cause programmed cell death (Quan et al., 2008).

MicroRNAs (miRNAs) are small, non-coding RNAs that have been demonstrated to be involved in many responses to and defenses against various biotic and abiotic stresses in plants (Bartel, 2004). With the development of high-throughput sequencing technology and bioinformatics, many plant miRNAs have been identified. In animals, at least 60% of protein-coding genes can be regulated by miRNAs (Friedman et al., 2009), but known target genes of miRNAs in plants are far fewer (~1% of the protein-coding genes) (Addo-Quaye et al., 2008; Li et al., 2010). Even so, the regulatory role of miRNAs in plants cannot be underestimated, because most known target genes are transcription factors (TFs) (Jones-Rhoades and Bartel, 2004; Jones-Rhoades et al., 2006). Under environmental stresses, plants up- or down-regulate certain miRNAs or synthesize new miRNAs to respond to or defend against stresses (Khraiwesh et al., 2012). Abiotic stresses, such as high light, UV, heat, heavy metals, drought, and salinity, can elevate ROS levels (Mittler et al., 2004). To date, only a few studies have focused on oxidative stress-triggered miRNA expression changes. miR398, whose target genes are Cu-Zn superoxide dismutases, is a well-studied miRNA related to the response to oxidative stress triggered by high light (Sunkar et al., 2006). Iyer et al. (2012) identified 22 ozone-induced oxidative stress miRNA families using a plant miRNA array in *Arabidopsis*; most of them were also reported as UV-B responsive miRNAs (Zhou et al., 2007). Jia et al. (2009) identified 24 UV-B responsive miRNAs (13 upregulated and 11 downregulated) in *Populus tremula* through a miRNA filter array. Some of these upregulated miRNAs (miR156, miR160, miR165/166, miR167, miR398, and miR168) were also reported in UV-B-stressed *Arabidopsis* (Zhou et al., 2007). Similarly, six miRNAs were identified as UV-B-responsive miRNAs in wheat (Wang et al., 2013), in which miR159, miR167a, and miR171 are upregulated and miR156, miR164, miR395 are downregulated. As a ROS, H_2_O_2_ can also act as a secondary messenger during stress response and defense signal transduction. However, only a few H_2_O_2_-responsive miRNAs (miR169, miR397, miR1425, miR408-5p, miR827, miR528, and miR319a.2) have been identified in plants (Li et al., 2011). Thus, the miRNAs related to oxidative stress caused by abiotic stressors are far from being completely elucidated in plants.

*Brachypodium distachyon*, as a model plant for crops such as wheat and barley, has been sequenced (Vogel et al., 2010). Several studies under abiotic stress have been performed at different levels, including the transcriptome (Priest et al., 2014), proteome, and phosphoproteome (Lv et al., 2014b). In addition, recent miRNA studies using high-throughput sequencing have identified many stress responsive miRNAs under several kinds of abiotic stress, such as cold stress (Zhang et al., 2009), dehydration stress (Budak and Akpinar, 2011), and drought stress (Bertolini et al., 2013). In particular, Jeong et al. (2013) sequenced 17 small RNA libraries that represented different tissues and stressors and identified many previously unreported and *B. distachyon*-specific miRNAs. However, the identified miRNAs are far from sufficient for *B. distachyon*, especially for oxidative stress regulated miRNAs.

Thus, in this study, we identified miRNAs and their potential target genes related to H_2_O_2_ stress using high-throughput sequencing, reverse transcription quantitative polymerase chain reaction (RT-qPCR) and 5′ RACE, combined with bioinformatics methods. The differentially expressed miRNAs observed between *B. distachyon* seedlings grown under control and H_2_O_2_-treated conditions, as well as the miRNA-directed regulatory network, provide new insights that will inform the genetic improvement of stress tolerance in plants.

## Materials and methods

### Plant materials

Seedlings of *B. distachyon* 21 (Bd21) were grown in a growth chamber at 25/20°C (16 h day/8 h night) and 70% relative humidity, as reported previously (Lv et al., 2014a). For H_2_O_2_ treatment, seedlings at the three leaves stage were treated with 20 mM H_2_O_2_ for 6 h in plastic containers and collected at 2, 4, or 6 h based on our previous study (Bian et al., 2015). Untreated seedlings were used as a control. All of the H_2_O_2_treated and untreated samples had three biological replicates and at least 100 seedlings were used in each replicate. All samples were snap-frozen in liquid nitrogen and then stored at −80°C until RNA extraction.

### Total RNA isolation

Total RNA was extracted from the frozen seedlings with TRIzol reagent (Invitrogen, Carlsbad, CA, USA) according to the manufacturer's instructions. Prior to nucleic precipitation, two extra chloroform washes were performed. A 1% agarose gel stained with ethidium bromide was run to determine the preliminary integrity of the RNA. All RNA samples were quantified and examined using an ND 1000 spectrophotometer (NanoDrop Technologies, Wilmington, DE, USA) for contamination with either protein (A260/A280 ratios) or reagent (A260/A230 ratios). The RNA integrity number (RIN) was >8, as determined with a 2100 Bioanalyzer (Agilent Technologies, Santa Clara, CA, USA).

### Construction of small RNA (sRNA) libraries and deep sequencing

For sRNA library construction and deep sequencing, RNA samples were prepared as follows: equal quantities (10 mg) of total RNA isolated from Bd21 seedlings treated with 20 mM H_2_O_2_ for 2, 4, and 6 h were mixed together to construct the TS library, and 30 mg of total RNA prepared from the control sample (without H_2_O_2_ treatment) were used to construct the CS library. Then, total RNA was separated by 15% TBE-urea denaturing polyacrylamide gel electrophoresis (PAGE), and RNA molecules in the range of 18–30 nt were enriched and ligated with proprietary adapters to the 5′ and 3′ termini by T4 RNA ligase. The samples were used as templates for cDNA synthesis by Super-Script II Reverse Transcriptase (Invitrogen) and the resulting cDNA was amplified to produce sequencing libraries. The final quality of the cDNA library was ensured by examining its size, purity, and concentration with a 2100 Bioanalyzer. The sequencing was performed by the Beijing Genomics Institute (BGI, Shenzhen, China). The two libraries were run on the Illumina HiSeq™ 2000 platform side by side.

### Bioinformatic analysis of sequencing data

After trimming the 30-bp adaptor sequence, sequences shorter than 18 nt were excluded from further analysis. First, rRNA, scRNA, snoRNA, snRNA, and tRNA in clean reads were identified by a blastall search against the Rfam (version 10.1) database. Next, sequences were perfectly mapped onto the Bd21 genome v1.0 (http://www.phytozome.net/) using the program SOAP2 (Li et al., 2009). Known miRNAs were identified according to *B. distachyon* defined mature miRNAs and stem-loop miRNA precursors from miRBase (version 20; http://www.mirbase.org) (Kozomara and Griffiths-Jones, 2011). Potential novel miRNAs were identified using the MIREAP (Li et al., 2012) software (http://sourceforge.net/projects/mireap/) based on Meyers et al. (2008), and unique sequences that had more than 10 hits to the genome or matches to known non-coding RNAs were removed. The secondary structures of novel miRNA precursors were predicted by RNAfold (http://rna.tbi.univie.ac.at/cgi-bin/RNAfold.cgi; Zuker, 2003) with default parameters.

### Differential expression analysis

The relative miRNA expression levels of the two libraries were compared and the differentially expressed miRNAs were screened based on a previously established method (Audic and Claverie, 1997). The frequency of miRNAs in the two libraries was normalized to one million by the total number of miRNAs in each library (transcripts per million (TPM) normalized expression = initial miRNA count^*^1,000,000/total count of clean reads). Following normalization, if the miRNA gene expression in both libraries was zero, then it was revised to 0.01; if the miRNA gene expression in both libraries was less than 1, owing to its too low expression, it was excluded from further differential expression analysis. Fold change = log2(the normalized H_2_O_2_ treatment reads/the normalized control reads). The *P*-value was calculated as described previously (Wu et al., 2014).

### Target gene prediction of miRNAs and functional analysis

Target genes were predicted using the MIREAP program developed by the BGI, combined with psRNATarget online software (http://www.plantgrn.org/psRNATarget/) (Dai and Zhao, 2011), and obeying the rules described in Allen et al. (2005) and Schwab et al. (2005). The criteria for using MIREAP and psRNATarget followed a previous study (Bertolini et al., 2013). Only the shared predictions of the two softwares were considered as the final target genes. The biological processes, molecular functions, and cellular components of the target genes were examined using the agriGO online tool (Du et al., 2010) to perform Gene Ontology (GO) annotation and GO enrichment analysis. The statistical test method was set as Fisher and the multi-test adjustment method was set as Bonferroni. The threshold of significance was defined as *p* < 0.01 and the false discovery rate (FDR) as < 0.01. The dataset containing protein sequences of *B. distachyon* genome was set as the background dataset.

### H_2_O_2_-responsive miRNA-mediated network analysis

The Search Tool for the Retrieval of Interacting Genes/Proteins (STRING) database of physical and functional interactions (Szklarczyk et al., 2011) was used to analyse the protein-protein interactions (PPI) of the proteins encoded by target genes of differentially expressed miRNAs. The miRNA-regulated PPI network was displayed by the Cytoscape (version 3.1.1) software (Shannon et al., 2003).

### Expression validation of miRNAs and their targets

We verified the patterns of expression of seven conserved *B. distachyon* miRNAs (miR159a-3p, miR159b-3p.1, miR160a/b/c/d-5p, miR169b, miR169d, miR397a, and miR528-5p). A miRcute miRNA First-strand cDNA Synthesis Kit (TIANGEN) was used for the RT reactions. The thermocycling program was adjusted to run for 60 min at 37°C, 5 s at 85°C, and then 4°C forever. For each miRNA, three biological replicates were used. The miR168 and 5.8S genes served as the endogenous controls (Bertolini et al., 2013; Jeong et al., 2013). All primers are listed in Table S1. RT-qPCR was conducted on a CFX96 Real-Time PCR Detection System (Bio-Rad). Each reaction included 2 μL of product from the diluted RT reactions, 1.0 μL of each primer (forward and reverse), 12.5 μL of SYBR® Premix Ex Taq™ (Perfect Real Time; TaKaRa), and 8.5 μL of nuclease-free water. The reactions were incubated in a 96-well plate at 95°C for 30 s, followed by 40 cycles of 95°C for 5 s, 60°C for 30 s, and 72°C for 10 s. All reactions were run in triplicate for each sample. All data were analyzed using the CFX Manager software (Bio-Rad). We also selected 13 target genes to validate their expression profiles in the CS and TS libraries via RT-qPCR following the method described by Lv et al. (2014b) according to the Minimum Information for Publication of Quantitative Real-Time PCR Experiments (MIQE) guidelines. The *Actin* and *SamDC* genes served as the endogenous controls (Lv et al., 2014b). All primers are listed in Table S1. Statistical analysis was performed using the SPSS 17.0 software. Statistical differences amongst the two libraries were assessed using the independent two-sample *t*-test. *P* < 0.05 were considered statistically significant.

### Modified RNA ligase-mediated (RLM) 5′ race for the mapping of mRNA cleavage sites

To identify cleavage sites in the target mRNAs, a modified RLM-5′-RACE was performed using a FirstChoice RLM-RACE Kit (Ambion, Austin, TX, USA). All the steps followed the manufacturer's instructions, except that the calf intestinal phosphatase treatment was omitted to maintain the cleaved transcripts. Nested PCR amplifications were performed using the general sense primers and gene specific nested antisense primers that were listed in Table S1. The amplification products were gel purified, cloned, and sequenced, and 10 independent clones were sequenced.

## Results

**Figure 1:**
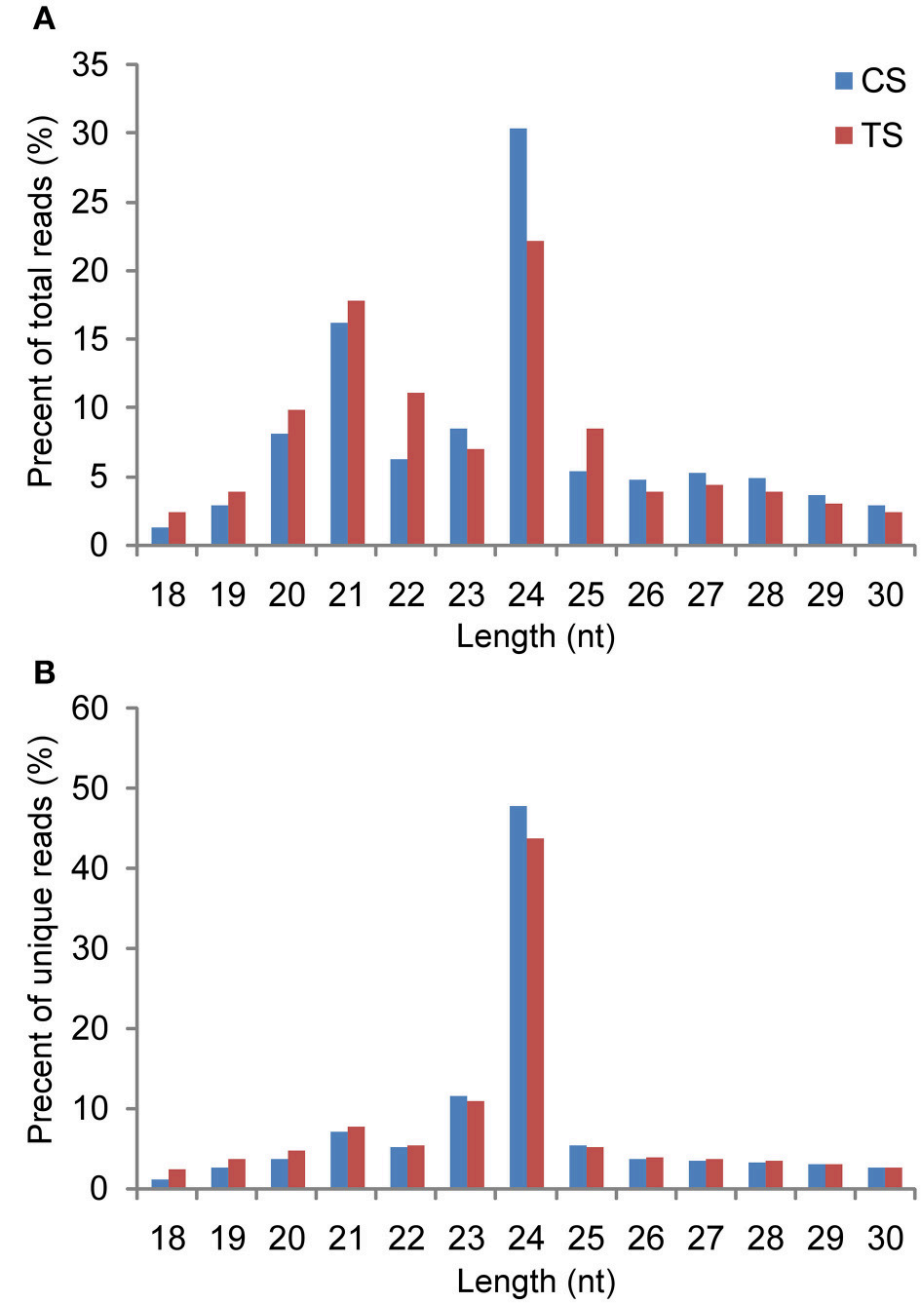
**Length distribution of sRNAs detected in the control library and stress library. (A)** Redundant sRNAs; **(B)** unique sRNAs.

In this work, the Illumina Solexa sequencing platform was used to investigate the genome-wide identification and expression profiles of miRNAs in *B. distachyon* under H_2_O_2_ stress. Two sRNA libraries were constructed using total RNAs isolated from control seedlings (CS) and H_2_O_2_-treated seedlings (TS). sRNA sequencing yielded a total of 18,220,106 and 19,373,978 high-quality raw sequence reads from the CS and TS libraries, respectively. The raw reads of the two libraries were uploaded to the National Center for Biotechnology Information (NCBI) Sequence Read Archive (SRA; accession numbers: SRX1542485 and SRX1542460). After removing low quality reads, adapters, poly-A sequences, and short RNA reads smaller than 18 nucleotides (nt), 17,811,109 (97.76%) and 17,708,762 (91.40%) clean reads representing 3,830,474 and 3,548,088 unique sRNAs were obtained from the CS and TS libraries, respectively (Table S2). Among the unique sequences, 1,963,866 (51.27%) and 1,689,996 (47.63%) generated from the CS and TS libraries, respectively, were mapped to the *B. distachyon* genome using SOAP2. To reveal the sequence distribution of the sRNAs, all clean reads were queried against the *B. distachyon* genome database at Phytozome (http://www.phytozome.net/), Rfam (http://rfam.sanger.ac.uk/), and miRBase v20.0 (http://www.mirbase.org/), and classified into seven annotation categories: non-coding RNAs (tRNA, rRNA, snRNA, and snoRNA), miRNA, exon-sense, exon-antisense, intron-sense, intron-antisense, and unknown sRNAs (Table S2). The length distribution of the total sRNA reads revealed that the majority of reads from each library were 20–25 nt in length, of which, 24-nt reads were the most abundant, followed by 21-nt reads (Figure 1A). Compared to the CS library, the TS library contained more 21-nt sRNAs and fewer 24-nt sRNAs. Of the unique sRNAs, 24-nt sRNAs accounted for 47.91 and 43.78% in the CS and TS libraries, respectively, while 21-nt sRNAs accounted for 5.10 and 5.33% (Figure 1B).

### Identification of known and novel miRNAs in *B. distachyon*

To identify known miRNAs from the CS and TS libraries, sRNA sequences generated from each library were independently aligned with currently known and experimentally validated mature *B. distachyon* miRNAs deposited in miRBase v20.0. Finally, a total of 144 known miRNAs were identified from the CS (133) and TS (138) libraries (Table S3); among them, 127 (88.19%) were detected in both libraries. For the total reads of each known miRNA in our study, 54.86% (79 miRNAs) had more than 100 reads, 29.17% (42) had more than 1000 reads, 12.5% (18) had more than 10,000 reads, and only 3.47% (5) had more than 100,000 reads. Among them, bdi-miR168-5p possessed the highest expression level in each library (556,771 reads in the CS library and 571,620 reads in the TS library), followed by bdi-miR156c, bdi-miR156b-5p, bdi-miR156d-5p, and bdi-miR528-5p.

After identifying the known miRNAs, the remaining sequences from the two libraries, which were classified as “unannotated” (excluding known miRNAs and other non-coding RNAs classified by Rfam), were used to discover novel and potential *B. distachyon*-specific miRNA candidates. To accomplish this, these small RNA sequences were aligned with the *B. distachyon* genome to identify genomic regions potentially harboring pre-miRNA sequences, whose hairpin-like structures are widely used to distinguish miRNAs from other small non-coding RNAs. The minimum free energy (MFE) of the secondary structures was also considered as a criterion for the prediction of potential pre-miRNAs. After aligning these unannotated sequences to the genome, we obtained a total of 221 novel miRNA candidates from the CS (156) and TS (132) libraries (Table S4). Among the 221 novel miRNA candidates, 36 had homologs in other plant species or their pre-miRNA sequences belonged to known *B. distachyon* pre-miRNAs (Table S4), while the remaining 185 novel miRNA candidates were *B. distachyon*-specific (Table S4). In agreement with previously reports, the cytosine and uracil nucleotides were dominant in the first position of the 5′ end for the majority of these newly determined putative novel miRNAs (Figure S1A). In detail, first positions included 11,629 cytosine nucleotides (67.52%) and 4279 uracil nucleotides (24.85%) in the CS library and 16,115 cytosine nucleotides (78.05%) and 3455 uracil nucleotides (16.73%) in the TS library. The first nucleotide bias analysis showed that cytosine was the most frequently used first nucleotide for novel 21-nt miRNAs and uracil was most frequent for novel 20-, 22-, and 23-nt miRNAs (Figure S1B). Our sequence analyses of the two libraries showed that the putative pre-miRNAs of each library greatly varied in length from 69 to 361 nt in the CS library and from 65 to 372 nt in the TS library. These pre-miRNA sequences were applied to predict the characteristic stem-loop secondary structure of pre-miRNA and their locations were also determined in the genomic loci (Table S4). We also calculated the minimum folding free energies of putative miRNA precursors for each library, which ranged from −18.1 to −180.64 kcal/mol with an average of −62.07 kcal/mol for the CS library and from −19.9 to −192.4 kcal/mol with an average of −60.46 kcal/mol for the TS library (Table S4). In contrast with the known miRNAs, most of the predicted novel miRNAs were expressed at very low levels. Only 9.50% (21) of the 221 novel miRNAs had more than 100 reads, and only two (novel_mir_59 and novel_mir_54) had more than 1000 reads (Table S4).

### Target gene prediction and GO annotation analysis

A total of 352 putative known miRNA target transcripts (corresponding to 284 target genes) and 554 putative novel miRNA target transcripts (corresponding to 460 target genes) were obtained (Table S5). The number of targets for each known miRNA and novel miRNA varied, ranging from 1 to 54 and 1 to 61, respectively, and the percent of novel miRNAs with more than 10 predicted target transcripts was 33.08%, while that number was 7.34% for known miRNAs. For comprehensive annotation, all putative target genes were analyzed by GO terms with the aid of the Blast2GO program with default parameters. Genes with a known function were categorized by biological process, molecular function, and cellular component according to the ontological definitions of the GO terms (Figure S2). For biological process, genes were mainly in the single-organism process (10.12%), response to stimulus (9.97%), localization (6.49%), multicellular organismal process (6.17%), and developmental process (6.01%) categories. For molecular function categories, nucleic acid binding transcription factor activity (4.11%), transporter activity (3.01%), and structural molecule activity (1.42%) were highlighted. For cellular component, they were mainly localized in the organelle (26.90%), membrane-bounded organelle (25.00%), and membrane (13.61%).

### Screening of H_2_O_2_-responsive miRNAs

**Figure 2:**
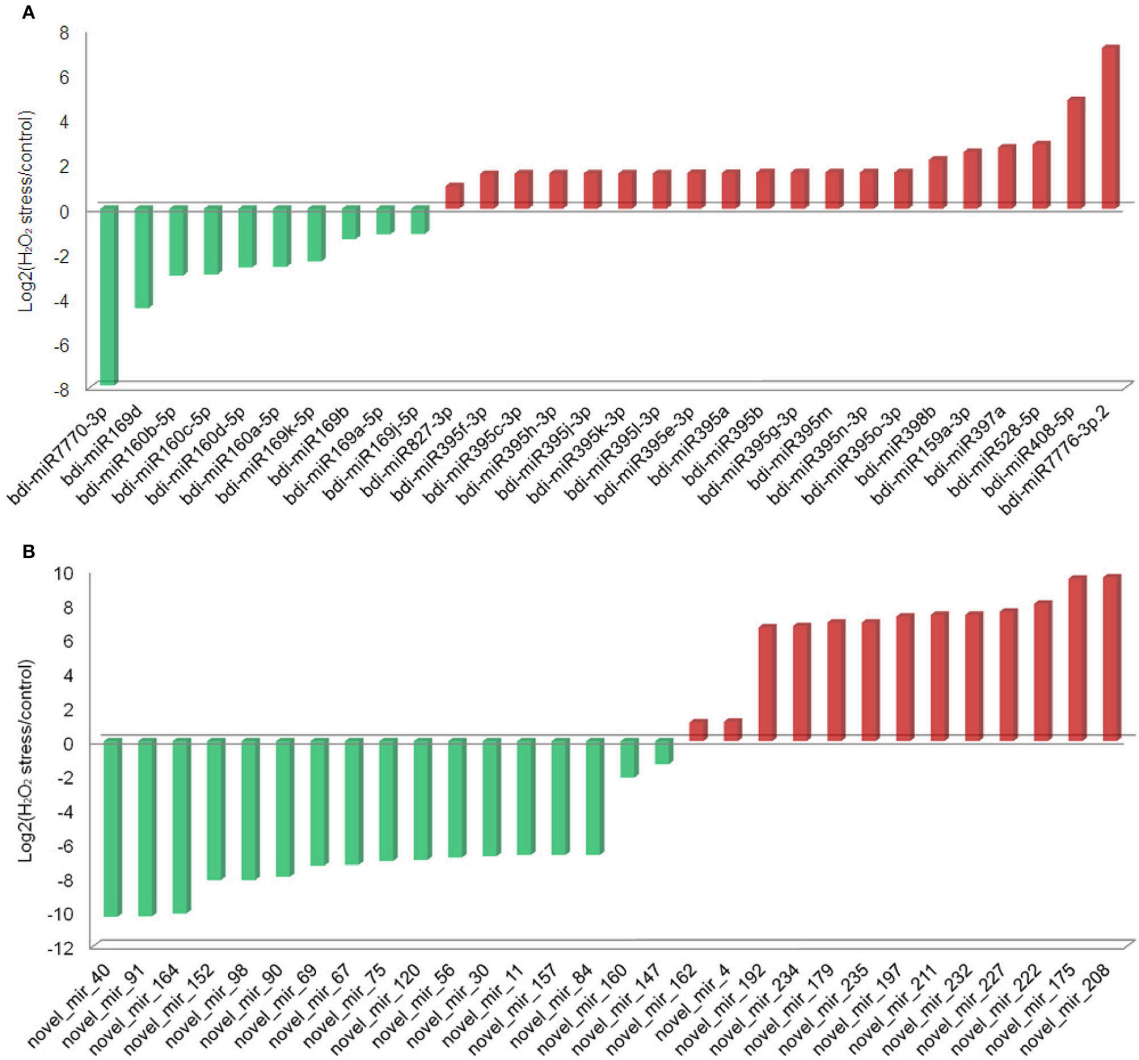
**Known and novel miRNAs differentially expressed between the CS library and TS library. (A)** Known miRNAs; **(B)** novel miRNAs.

In this study, 31 known and 30 novel miRNAs were observed with a more than two-fold change in response to H_2_O_2_ treatment in *B. distachyon* seedlings (Figure 2, Tables 1, 2). As reported in a previous study (Li et al., 2011), a series of known H_2_O_2_-responsive miRNAs, including miR159, miR160, miR169, miR397, and miR528, were also identified in our study. Further analysis revealed that 10 of the 31 known miRNAs were downregulated in the TS library compared to the CS library, whereas 21 were upregulated (Figure 2A). The 10 downregulated known miRNAs were composed of three miRNA families, including five members of miR169, four members of miR160, and miR7770. Among them, miR7770 displayed a dramatic (log2 fold change = −7.92) decrease. For the 21 upregulated known miRNAs, miR395 was the major family containing 14 members. bdi-miR159b-3p.1 showed the highest upregulation (log2 fold change = 16.21).

**Table 1:** **Differential expressed known miRNA between CS and TS libraries**.

**Sequence 5′–3′**	**miR-name**	Log2(T/C)^*^	***p*-value**	**Target gene**	**Putative function of targets**
UCUAGACGGGCCUUCAAAGAG	bdi-miR7770-3p	−7.92	1.29E−13	Bradi3g49427	Regulator of non-sense transcripts UPF2
				Bradi5g25910	Unknown
UAGCCAAGAAUGACUUGCCUA	bdi-miR169d	−4.45	3.18E−06	Bradi4g01380	Nuclear transcription factor Y subunit A-2
				Bradi1g11800	Nuclear transcription factor Y subunit A-4
				Bradi3g57320	Nuclear transcription factor Y subunit A-4
UGCCUGGCUCCCUGUAUGCCA	bdi-miR160b-5p	−2.99	8.83E−11	Bradi3g28950	Auxin response factor 22
UGCCUGGCUCCCUGUAUGCCA	bdi-miR160c-5p	−2.94	2.79E−10	Bradi3g28950	Auxin response factor 22
UGCCUGGCUCCCUGUAUGCCA	bdi-miR160d-5p	−2.63	1.36E−09	Bradi3g28950	Auxin response factor 22
UGCCUGGCUCCCUGUAUGCCA	bdi-miR160a-5p	−2.60	2.35E−09	Bradi3g28950	Auxin response factor 22
UAGCCAAGGAUGAUUUGCCUGU	bdi-miR169k-5p	−2.36	1.67E−13	Bradi3g57320	Nuclear transcription factor Y subunit A-4
				Bradi1g11800	Nuclear transcription factor Y subunit A-4
UAGCCAAGGAUGACUUGCCGG	bdi-miR169b	−1.36	8.38E−16	Bradi3g57320	Nuclear transcription factor Y subunit A-4
CAGCCAAGGAUGACUUGCCGA	bdi-miR169a-5p	−1.14	5.53E−57	Bradi3g57320	Nuclear transcription factor Y subunit A-4
				Bradi2g58570	Protein YLS7
UAGCCAGGAAUGGCUUGCCUA	bdi-miR169j-5p	−1.13	5.81E−09	NA	
UUAGAUGACCAUCAGCAAACA	bdi-miR827-3p	1.01	3.29E−10	Bradi1g62277	Unknown
UGAAGUGUUUGGGGGAACUC	bdi-miR395f-3p	1.56	8.32E−13	Bradi1g09030	ATP sulfurylase 1
				Bradi1g24110	Ribosome-recycling factor
				Bradi2g52840	Disease resistance protein RGA4
UGAAGUGUUUGGGGGAACUC	bdi-miR395c-3p	1.58	6.27E−13	Bradi1g09030	ATP sulfurylase 1
				Bradi1g24110	Ribosome-recycling factor
				Bradi2g52840	Disease resistance protein RGA4
UGAAGUGUUUGGGGGAACUC	bdi-miR395h-3p	1.58	6.27E−13	Bradi1g09030	ATP sulfurylase 1
				Bradi1g24110	Ribosome-recycling factor
				Bradi2g52840	Disease resistance protein RGA4
UGAAGUGUUUGGGGGAACUC	bdi-miR395j-3p	1.58	6.27E−13	Bradi1g09030	ATP sulfurylase 1
				Bradi1g24110	Ribosome-recycling factor
				Bradi2g52840	Disease resistance protein RGA4
UGAAGUGUUUGGGGGAACUC	bdi-miR395k-3p	1.58	6.27E−13	Bradi1g09030	ATP sulfurylase 1
				Bradi1g24110	Ribosome-recycling factor
				Bradi2g52840	Disease resistance protein RGA4
UGAAGUGUUUGGGGGAACUC	bdi-miR395l-3p	1.58	6.27E−13	Bradi1g09030	ATP sulfurylase 1
				Bradi1g24110	Ribosome-recycling factor
				Bradi2g52840	Disease resistance protein RGA4
UGAAGUGUUUGGGGGAACUC	bdi-miR395e-3p	1.6	4.70E−13	Bradi1g09030	ATP sulfurylase 1
				Bradi1g24110	Ribosome-recycling factor
				Bradi2g52840	Disease resistance protein RGA4
UGAAGUGUUUGGGGGAACUC	bdi-miR395a	1.6	8.05E−13	Bradi1g09030	ATP sulfurylase 1
				Bradi1g24110	Ribosome-recycling factor
				Bradi2g52840	Disease resistance protein RGA4
UGAAGUGUUUGGGGGAACUC	bdi-miR395b	1.63	2.30E−13	Bradi1g09030	ATP sulfurylase 1
				Bradi1g24110	Ribosome-recycling factor
				Bradi2g52840	Disease resistance protein RGA4
UGAAGUGUUUGGGGGAACUC	bdi-miR395g-3p	1.63	2.30E−13	Bradi1g09030	ATP sulfurylase 1
				Bradi1g24110	Ribosome-recycling factor
				Bradi2g52840	Disease resistance protein RGA4
UGAAGUGUUUGGGGGAACUC	bdi-miR395m	1.63	2.30E−13	Bradi1g09030	ATP sulfurylase 1
				Bradi1g24110	Ribosome-recycling factor
				Bradi2g52840	Disease resistance protein RGA4
UGAAGUGUUUGGGGGAACUC	bdi-miR395n-3p	1.63	2.30E−13	Bradi1g09030	ATP sulfurylase 1
				Bradi1g24110	Ribosome-recycling factor
				Bradi2g52840	Disease resistance protein RGA4
UGAAGUGUUUGGGGGAACUC	bdi-miR395o-3p	1.63	2.30E−13	Bradi1g09030	ATP sulfurylase 1
				Bradi1g24110	Ribosome-recycling factor
				Bradi2g52840	Disease resistance protein RGA4
CAGGAGUGUCACUGAGAACACA	bdi-miR398b	2.2	1.14E−20	Bradi3g43220	Unknown
CUUGGAUUGAAGGGAGCUCU	bdi-miR159a-3p	2.54	2.08E−05	Bradi2g53010	Transcription factor GAMYB
				Bradi1g36540	Transcription factor GAMYB
UCAUUGAGUGCAGCGUUGAUG	bdi-miR397a	2.74	1.20E−278	Bradi1g24880	Laccase-4
				Bradi1g24910	Laccase-4
				Bradi1g66720	Laccase-10
				Bradi1g74320	Laccase-22
				Bradi2g23350	Laccase-12/13
				Bradi2g54680	Laccase-4
				Bradi2g54690	Laccase-12/13
				Bradi2g55060	Laccase-8
				Bradi3g03407	Auxin response factor 5
UGGAAGGGGCAUGCAGAGGAG	bdi-miR528-5p	2.88	0	Bradi3g19170	E3 ubiquitin-protein ligase XBOS35
				Bradi1g54580	Endoglucanase 19
CAGGGAUGGAGCAGAGCAUGG	bdi-miR408-5p	4.87	0	Bradi1g45220	Transcription factor TCP15
				Bradi4g09417	Xylanase inhibitor protein 1
UUGAUAAUGGGUUGAAUGCGC	bdi-miR7776-3p.2	7.2	1.38E−08	NA	
UUUGGAUUGAAGGGAGCUCUG	bdi-miR159b-3p.1	16.21	0	Bradi2g53010	Transcription factor GAMYB
				Bradi1g36540	Transcription factor GAMYB
				Bradi1g60120	MOB kinase activator 1
				Bradi3g52980	Histidinol-phosphate aminotransferase

* *Log2(T/C): Log2(H_2_O_2_ treatment/control)*.

**Table 2:** **Differential expressed novel miRNA between CS and TS libraries**.

**Sequence 5′–3′**	**miR-name**	Log2(T/C)^*^	***p*-value**	**Target gene**	**Putative function of targets**
GCAGGUUGUUCUUGGCUAACA	novel_mir_40	−10.28	2.82E−67	Bradi1g25517	Glucan endo-1,3-beta-glucosidase 3
AGAAUUUAGGAUCGGAAGGAG	novel_mir_91	−10.26	4.46E−66	Bradi1g32850	CRIB domain-containing protein
				Bradi3g18240	3beta-hydroxysteroid-dehydrogenase/decarboxylase isoform 1
				Bradi1g58890	Unknown
				Bradi1g37377	U-box domain-containing protein 34
				Bradi5g24267	Dedicator of cytokinesis protein 8
				Bradi1g34327	L-arabinokinase
				Bradi4g11510	Sister-chromatid cohesion protein 3
				Bradi3g37170	Unknown
				Bradi2g55770	Unknown
				Bradi4g34470	Unknown
				Bradi2g07860	VHS domain-containing protein At3g16270
				Bradi1g43430	Unknown
				Bradi1g56500	BTB/POZ and MATH domain-containing protein 5
				Bradi1g76677	Myosin-9
				Bradi2g15960	Protein timeless homolog
				Bradi2g23290	Kinesin-like protein KIF22
				Bradi2g42100	V-type proton ATPase subunit d
				Bradi2g47267	Symplekin
				Bradi3g03910	Isoflavone 2′-hydroxylase
				Bradi3g05640	Proline-rich protein PRCC
				Bradi3g16880	Indole-3-glycerol phosphate lyase
				Bradi3g23160	Alanine aminotransferase 2
				Bradi3g47270	Unknown
				Bradi3g58750	Outer envelope protein 64
				Bradi5g19230	RING-H2 finger protein ATL81
CCCGGUCGAGGACGGCCCCGCC	novel_mir_164	−10.10	3.50E−59	Bradi1g20780	Phytoene dehydrogenase
UUAACGAGAGCACCAAUGACACC	novel_mir_152	−8.13	1.03E−15	Bradi1g06530	Cell differentiation protein RCD1 homolog
				Bradi1g69850	Cell differentiation protein RCD1 homolog
				Bradi5g21620	Cell differentiation protein rcd1
				Bradi1g58330	Cell differentiation protein rcd1
UUUUAUGGAUCAGAGGGAGUAU	novel_mir_98	−8.13	1.03E−15	Bradi4g10380	Unknown
				Bradi2g03701	Unknown
				Bradi1g14810	Zinc finger protein 7
				Bradi2g55497	Transcription initiation factor TFIID subunit 12
				Bradi5g18760	Methyltransferase PMT13
				Bradi3g52740	Soluble inorganic pyrophosphatase
				Bradi1g53320	IAA-amino acid hydrolase ILR1-like 7
				Bradi3g27520	Transcription factor-like protein DPB
AAAGAUUGGCAUGGAUUUGAA	novel_mir_90	−7.95	6.47E−14	Bradi3g39450	WD repeat-containing protein C2A9.03
				Bradi5g20726	Xyloglucan endotransglucosylase/hydrolase protein 13
				Bradi2g24715	2-oxoglutarate-dependent dioxygenase DAO
				Bradi3g54330	Unknown
UUUGAAUGUAUGUAGACAUGA	novel_mir_69	−7.30	4.05E−09	Bradi1g18990	Annexin D3
				Bradi2g58070	Vacuolar protein sorting-associated protein 53 A
				Bradi1g71570	Mitochondrial import inner membrane translocase subunit TIM22-2
				Bradi5g19590	Unknown
				Bradi5g24090	Filament-like plant protein 7
				Bradi3g35197	Unknown
				Bradi3g43737	DNA repair protein RAD50
UUCGAUUGCAAGAUGACAGGU	novel_mir_67	−7.24	8.08E−09	Bradi4g27820	Unknown
CACUUAUUAUCGAUCGGAGGG	novel_mir_75	−7.01	1.28E−07	NA	
ACUUAUUAUGGAUCGGAGGGG	novel_mir_120	−6.95	2.55E−07	Bradi3g52740	Soluble inorganic pyrophosphatase
				Bradi4g10380	Unknown
				Bradi4g20020	O-methyltransferase 2
				Bradi1g53320	IAA-amino acid hydrolase ILR1-like 7
				Bradi2g55497	Transcription initiation factor TFIID subunit 12
				Bradi4g31100	Unknown
				Bradi5g10310	Phosphoribosylglycinamide formyltransferase
				Bradi5g18760	Methyltransferase PMT13
				Bradi1g02840	BTB/POZ domain-containing protein POB1
				Bradi1g38687	Spermine synthase
				Bradi1g60350	Unknown
				Bradi1g61057	tRNA pseudouridine(38/39) synthase
				Bradi1g78570	DeSI-like protein At4g17486
				Bradi2g25327	Disease resistance protein RGA1
				Bradi2g27920	Ethylene-responsive transcription factor RAP2-3
				Bradi2g50870	Deoxycytidylate deaminase
				Bradi3g21077	Purine permease 3
				Bradi3g27520	Transcription factor-like protein DPB
				Bradi3g29950	Prolyl 4-hydroxylase 3
				Bradi3g34557	NADH dehydrogenase [ubiquinone] 1 alpha subcomplex subunit 12
AAGUAAUAUGGAUCGGAGGAAGU	novel_mir_56	−6.81	1.01E−06	Bradi2g24380	Unknown
				Bradi4g09330	Unknown
				Bradi2g27700	Thiamine pyrophosphokinase 3
				Bradi2g18170	Unknown
				Bradi3g29950	Prolyl 4-hydroxylase 3
				Bradi5g13500	Unknown
				Bradi2g25327	Disease resistance protein RGA1
				Bradi4g28270	Pleiotropic drug resistance protein 4
				Bradi2g34820	E3 ubiquitin-protein ligase UPL6
				Bradi4g39027	Unknown
AAGAGUAGCGUUGAUACACCGU	novel_mir_30	−6.74	2.02E−06	NA	
UACGUGAGUUAAAUCGUCGAC	novel_mir_11	−6.66	4.03E−06	NA	
ACUUAUUAUGAAUCGGAGGGG	novel_mir_157	−6.66	4.03E−06	Bradi4g38600	Unknown
				Bradi3g06880	Protein YIF1B
				Bradi3g45327	Unknown
				Bradi3g51490	Transcription factor EMB1444
				Bradi5g26806	Cleavage and polyadenylation specificity factor subunit 5
				Bradi3g15457	G-type lectin S-receptor-like serine/threonine-protein kinase B120
ACAUGAUAUGGAUGGUGAUGUG	novel_mir_84	−6.66	4.03E−06	NA	
CAUGGUAUUGUUUCGGCUCAUG	novel_mir_160	−2.12	5.72E−17	NA	
UCCCUCUCUCCCUUGAAGGCU	novel_mir_147	−1.35	6.42E−07	Bradi3g52690	DNA-binding protein SMUBP-2
GUUUCCUGCAAGCACUUCACG	novel_mir_162	1.1	2.35E−03	NA	
UUGACUUAAGACAAAGCUAG	novel_mir_4	1.15	6.85E−04	Bradi4g08140	SAGA-associated factor 29 homolog
				Bradi4g27070	Unknown
				Bradi1g43160	Very-long-chain 3-oxoacyl-CoA reductase 1
				Bradi2g25710	Cytochrome P450 734A1
				Bradi1g28650	Luc7-like protein 3
				Bradi4g38147	UDP-3-O-acylglucosamine N-acyltransferase 2
				Bradi1g46367	Omega-amidase
AGGAGCGAGACGGUAAGCCCU	novel_mir_192	6.67	3.61E−06	NA	
UGGACUGCAGGUUUAUUUCGG	novel_mir_234	6.75	1.80E−06	Bradi3g00227	Ferredoxin-thioredoxin reductase catalytic chain
				Bradi4g43410	Unknown
GAUUAAGAUCCGACGGCCAAACA	novel_mir_179	6.96	2.23E−07	NA	
AGAGUUGAUAACGGAUUCGGAUA	novel_mir_235	6.96	2.23E−07	Bradi3g26780	Acylamino-acid-releasing enzyme
				Bradi4g35630	CBF5
CAAGAAUUUAGGGACGGAGGG	novel_mir_197	7.3	3.43E−09	Bradi4g38600	Unknown
				Bradi1g34327	L-arabinokinase
				Bradi1g37377	U-box domain-containing protein 34
				Bradi1g58890	Unknown
				Bradi5g01850	WEB family protein At2g17940
				Bradi1g32850	CRIB domain-containing protein
				Bradi3g03910	Isoflavone 2′-hydroxylase
				Bradi1g50390	Unknown
				Bradi1g76677	Myosin-9
				Bradi2g05980	Cytochrome P450 90D2
				Bradi2g07860	VHS domain-containing protein At3g16270
				Bradi2g15960	Protein timeless homolog
				Bradi2g36370	Serine/threonine-protein phosphatase BSL1 homolog
				Bradi2g55770	Unknown
				Bradi3g37170	Unknown
				Bradi4g34470	Unknown
				Bradi5g04577	Unknown
				Bradi5g19230	RING-H2 finger protein ATL81
UGUGCACUUGGACCAAGACAGCU	novel_mir_211	7.4	8.52E−10	Bradi4g03757	3-ketoacyl-CoA synthase 6
				Bradi3g02120	Unknown
CGCGCGUCGCUGUCAAGGGG	novel_mir_232	7.4	8.52E−10	NA	
CGACAAGAAUUUAGGGACGGA	novel_mir_227	7.58	5.26E−11	Bradi3g03910	Isoflavone 2′-hydroxylase
				Bradi4g38600	Unknown
				Bradi1g50390	Unknown
				Bradi5g19230	RING-H2 finger protein ATL81
				Bradi3g46930	Cell number regulator 2
				Bradi5g25070	GTP cyclohydrolase I
				Bradi3g51260	Protein RMD5 homolog A
				Bradi1g34327	L-arabinokinase
				Bradi1g42810	Protein-tyrosine-phosphatase IBR5
				Bradi2g13670	Metal transporter Nramp4
				Bradi2g14200	Unknown
				Bradi2g31560	Unknown
				Bradi2g38827	Disease resistance protein RPM1
				Bradi2g51040	Factor of DNA methylation 1
				Bradi3g06402	Glycerophosphodiester phosphodiesterase GDPDL7
				Bradi3g31810	Unknown
				Bradi3g44030	Unknown
				Bradi3g47890	Unknown
				Bradi4g07420	DCN1-like protein 2
				Bradi5g01850	WEB family protein At2g17940
CAUUUAUUAUGGAUCGGAGGU	novel_mir_222	8.05	6.19E−15	Bradi3g52740	Soluble inorganic pyrophosphatase
				Bradi4g38600	Unknown
				Bradi1g53320	IAA-amino acid hydrolase ILR1-like 7
				Bradi4g10380	Unknown
				Bradi1g02840	BTB/POZ domain-containing protein POB1
				Bradi1g78570	DeSI-like protein At4g17486
				Bradi2g27920	Ethylene-responsive transcription factor RAP2-3
				Bradi2g55497	Transcription initiation factor TFIID subunit 12
				Bradi1g14810	Zinc finger protein 7
				Bradi1g39270	Ubiquitin-conjugating enzyme E2 5B
				Bradi1g60350	Unknown
				Bradi1g60890	Intron-binding protein aquarius
				Bradi2g25327	Disease resistance protein RGA1
				Bradi3g21077	Purine permease 3
				Bradi3g27520	Transcription factor-like protein DPB
				Bradi3g29950	Prolyl 4-hydroxylase 3
				Bradi4g31100	Unknown
				Bradi5g10310	Phosphoribosylglycinamide formyltransferase
AGGACCGGUGAAGGGGGCGGA	novel_mir_175	9.52	5.03E−40	Bradi1g30570	Unknown
				Bradi1g13320	Ankyrin repeat domain-containing protein 65
				Bradi3g06847	Brefeldin A-inhibited guanine nucleotide-exchange protein 1
AAUCGAGUAGCAGUCCGCGGU	novel_mir_208	9.6	3.85E−42	NA	

* *Log2(T/C): Log2(H_2_O_2_ treatment/control)*.

Among the 30 differential novel miRNAs, 17 downregulated and 13 upregulated miRNAs were found in the TS library compared to the CS library (Figure 2B). All but three novel miRNAs (novel_mir_4, novel_mir_148, and novel_mir_161) showed dramatic changes (log2 fold change >4 or < −4), in contrast to known differential miRNAs, of which only four exhibited dramatic changes.

**Figure 3:**
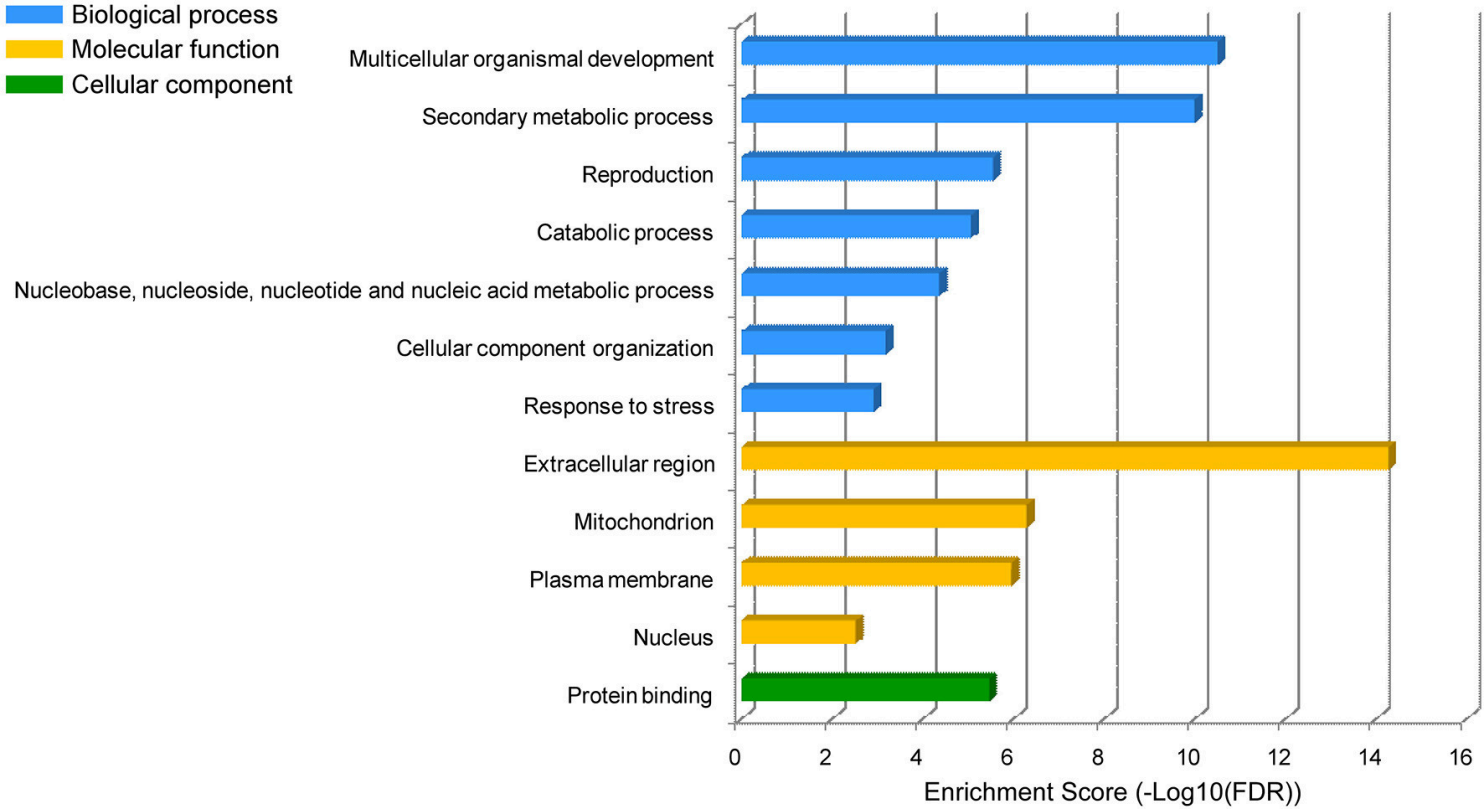
**GO enrichment of the H_2_O_2_-responsive miRNA target genes**. The dataset containing protein sequences of *B. distachyon* genome was set as the background dataset. The vertical axis is the enriched GO category, and the horizontal axis is GO enrichment.

We performed GO enrichment analysis for the target genes of these H_2_O_2_-responsive miRNAs and found that the proteins encoded by these target genes were mainly involved in the categories of multicellular organismal development (GO:0007275, FDR:3.1E−11), secondary metabolic process (GO:0019748, FDR:1.0E−10), reproduction (GO:0000003, FDR:2.8E−6), catabolic process (GO:0009056, FDR:8.6E−6), nucleobase, nucleoside, nucleotide and nucleic acid metabolic process (GO:0006139, FDR:4.3E−5), cellular component organization (GO:0016043, FDR:6.6E−4), and response to stress (GO:0006950, FDR:1.2E−3) (Figure 3). These proteins mainly exhibited binding function (GO:0005515, FDR:3.4E−6) and were localized in the extracellular region (GO:0005576, FDR:5.3E−15), mitochondrion (GO:0005739, FDR:5.1E−7), plasma membrane (GO:0005886, FDR:1.1E−6), and nucleus (GO:0005634, FDR:3.0E−3) (Figure 3).

### A miRNA-mediated regulatory network for H_2_O_2_ stress response

**Figure 4:**
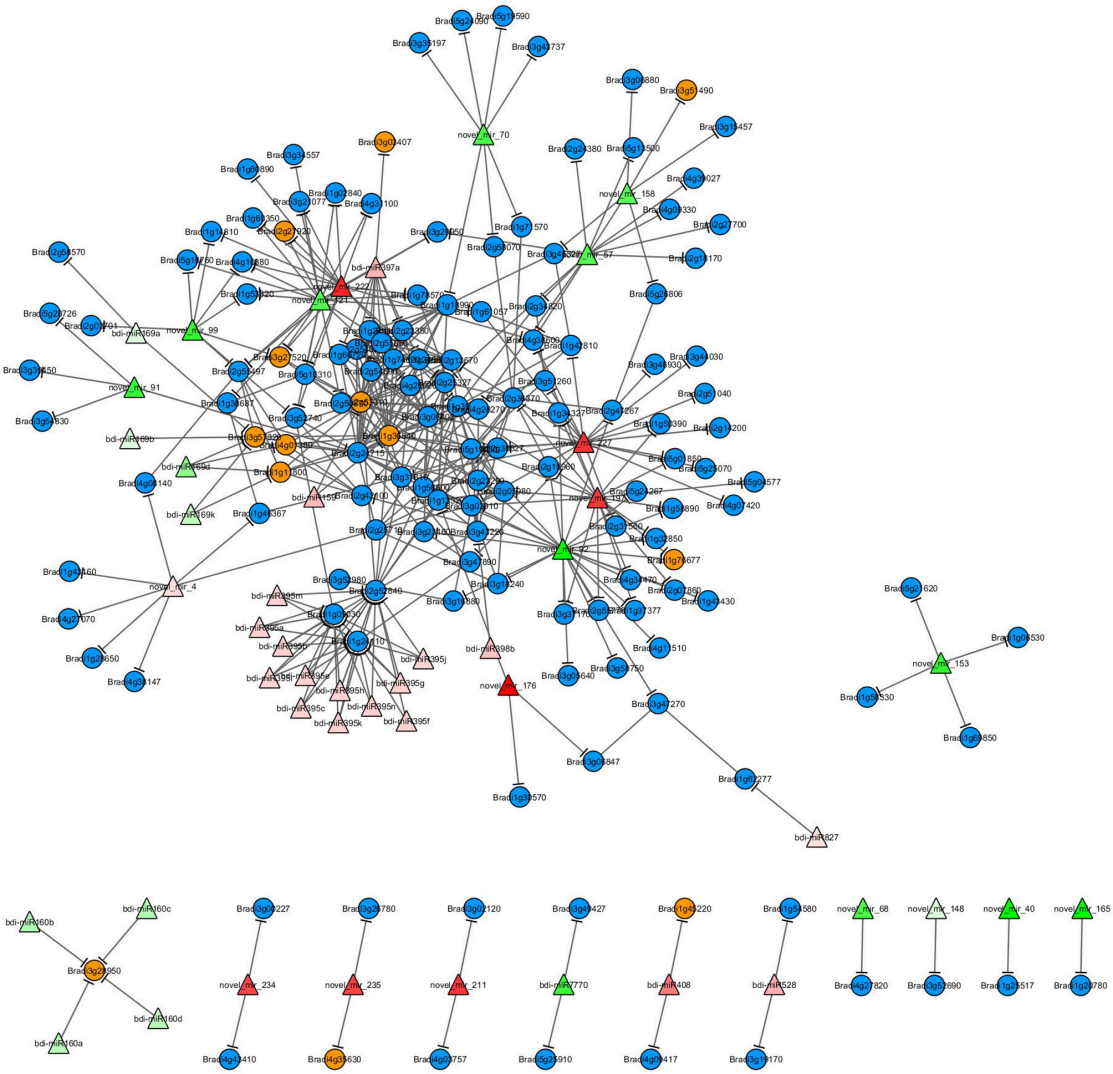
**A miRNA-mediated H_2_O_2_-responsive regulatory network**. Triangles represent the differentially expressed miRNAs and the color gradient shows the fold change (red: upregulation; green: downregulation). Blue circles represent the target genes of differentially expressed miRNAs; yellow circles represent target genes that encode transcription factors.

Based on the miRNA target prediction and protein-protein interaction (PPI) analysis, the H_2_O_2_-responsive miRNAs and the proteins encoded by their target genes were used to construct the miRNA-mediated regulatory network for H_2_O_2_ stress response (Figure 4). Generally, the more proteins that a protein interacts with, the more important are that protein's functions in the network. Two MYB TFs (encoded by genes *Bradi2g53010* and *Bradi1g36540*) were centered in the network and each could interact with 24 proteins. Thus, the two MYB TFs may play critical roles during H_2_O_2_-triggered oxidative stress response. Some target genes were regulated by two or more miRNAs that belonged to the same miRNA family or different families, and the proteins encoded by these genes always interacted with more proteins. For example, *Bradi2g52840* (target gene of the bdi-miR395 family) encodes disease resistance protein RGA4, which interacts with seven proteins encoded by the target genes of H_2_O_2_ stress-responsive miRNAs, including the two MYB TFs mentioned above (Figure 4). Gene *Bradi2g55497* is regulated by three novel miRNAs (novel_mir_98, novel_mir_120, and novel_mir_222) and encodes transcription initiation factor TFIID subunit 12, which can interact with four proteins, including three members of the NFYA TF family (encoded by genes *Bradi4g01380, Bradi1g11800*, and *Bradi3g57320*) and a transcriptional regulator SAGA-associated factor 29 homolog (encoded by gene *Bradi4g08140*).

### RT-qPCR validation of *B. distachyon* miRNAs and target genes

**Figure 5:**
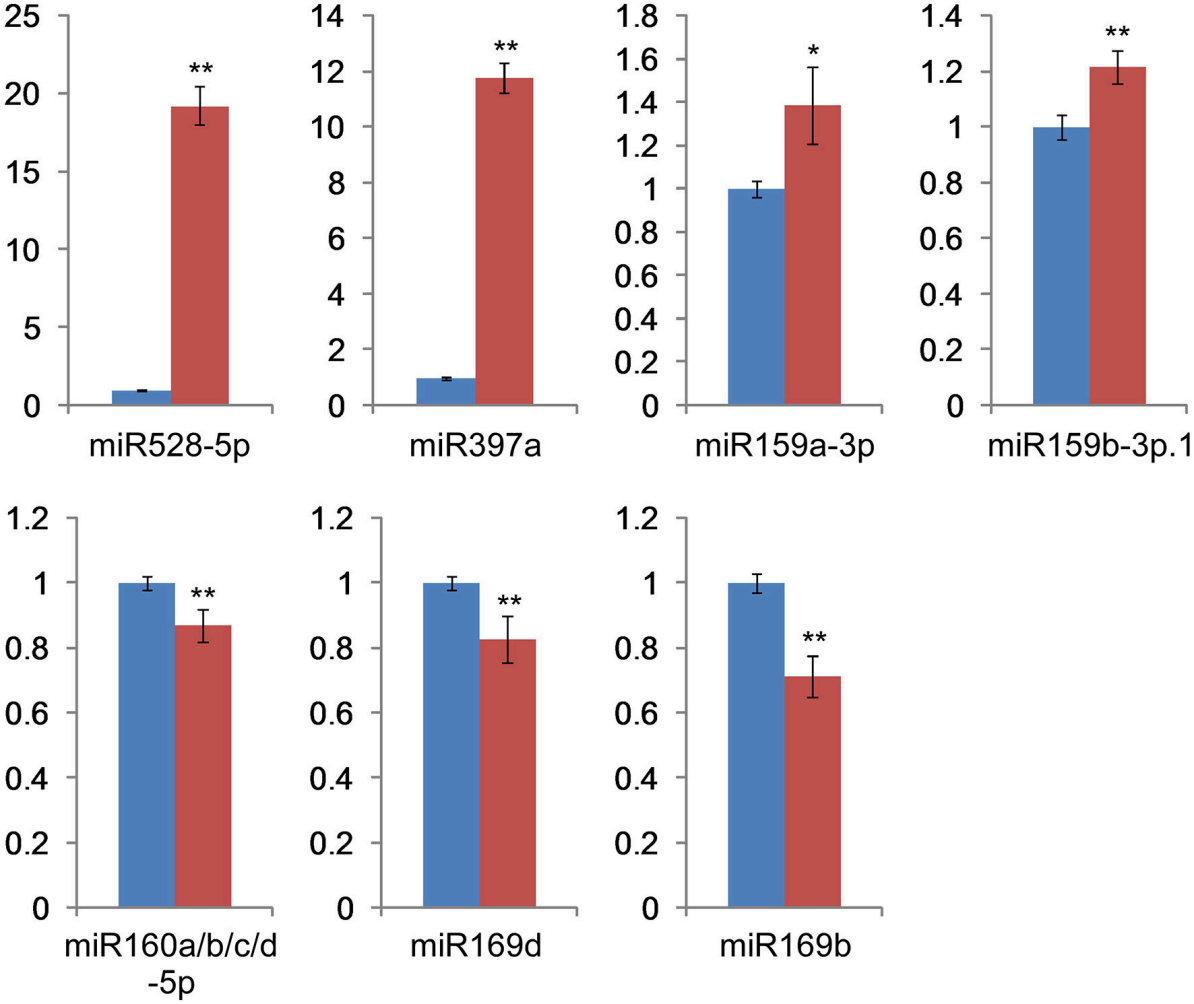
**RT-qPCR validation of the miRNAs**. Blue bar: relative gene expression level in the control library. Red bar: relative gene expression level in the H_2_O_2_ stress library. The data are derived from three biological repeats and represent mean ± standard deviation (*n* = 3). “^*^” and “^**^” indicate significant difference at *P* < 0.05 and 0.01 level, respectively.

We applied RT-qPCR analysis for further experimental verification of the presence of several conserved miRNAs and compared the expression patterns of these miRNAs with deep sequencing results. Analysis of seven H_2_O_2_-responsive miRNAs (miR159a-3p, miR159b-3p.1, miR160a/b/c/d-5p, miR169b, miR169d, miR397a, and miR528-5p) by RT-qPCR (Figure 5) showed that all of the relative expression profiles exhibited the same trends as their deep sequencing results, although there were some extent differences between the results obtained from deep sequencing and the RT-qPCR experiment. In detail, miR528-5p, miR397a, miR159a-3p, and miR159b-3p.1 were up-regulated under H_2_O_2_ stress, while the expression of miR160a/b/c/d-5p, miR169d, and miR169b decreased during H_2_O_2_ treatment.

**Figure 6:**
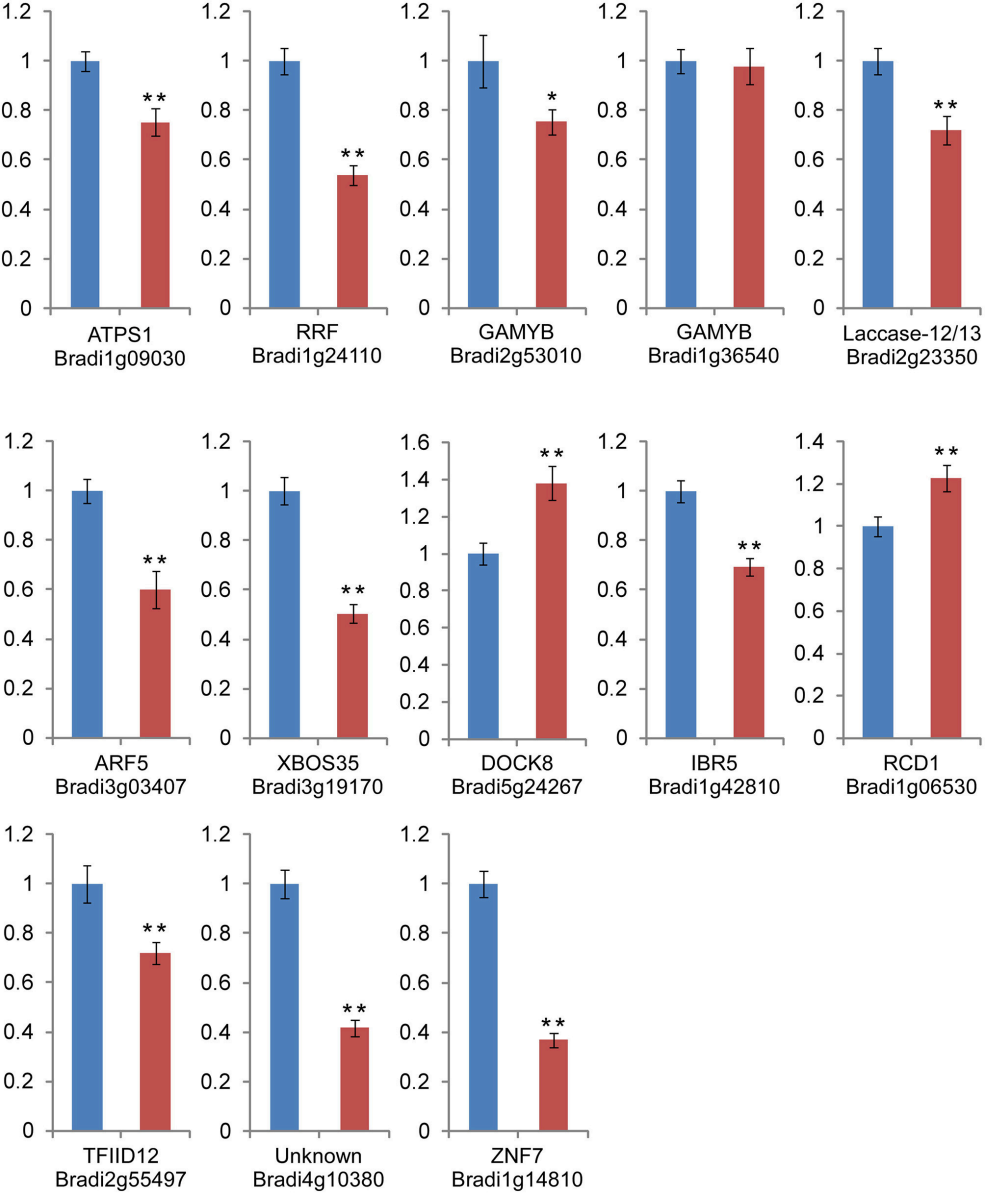
**RT-qPCR analysis of the miRNA target genes**. The *Actin* and *SamDC* genes were served as the endogenous controls. Blue bar: relative gene expression level in the control library. Red bar: relative gene expression level in the H_2_O_2_ stress library. The data are derived from three biological repeats and represent mean ± standard deviation (*n* = 3). “^*^” and “^**^” indicate significant difference at *P* < 0.05 and 0.01 level, respectively.

RT-qPCR was also used for detection and quantification of the predicted targets of 13 H_2_O_2_-responsive miRNAs (Figure 6). Our results revealed that for most of the miRNAs, there was a negative correlation between the miRNA level and the levels of their target genes (Figure 6), with the exception of *Bradi1g36540*. *Bradi1g36540* and *Bradi2g53010* were both regulated by the upregulated bdi-miR159a-3p and bdi-miR159b-3p.1. *Bradi2g53010* was downregulated, but *Bradi1g36540* showed no significant change under H_2_O_2_ stress. *Bradi2g55497* was mediated by three novel miRNAs (Figure 4). Two of the miRNAs (novel_mir_98 and novel_mir_120) were downregulated, while another (novel_mir_222) was upregulated, but the expression level of *Bradi2g55497* was unexpectedly downregulated. This phenomenon was also observed in the profiles of *Bradi4g10380* (the target gene of novel_mir_98, novel_mir_120, and novel_mir_222) and *Bradi1g14810* (the target gene of novel_mir_98 and novel_mir_222).

### Verification of miRNA-guided cleavage of target mRNAs in *B. distachyon*

**Figure 7:**
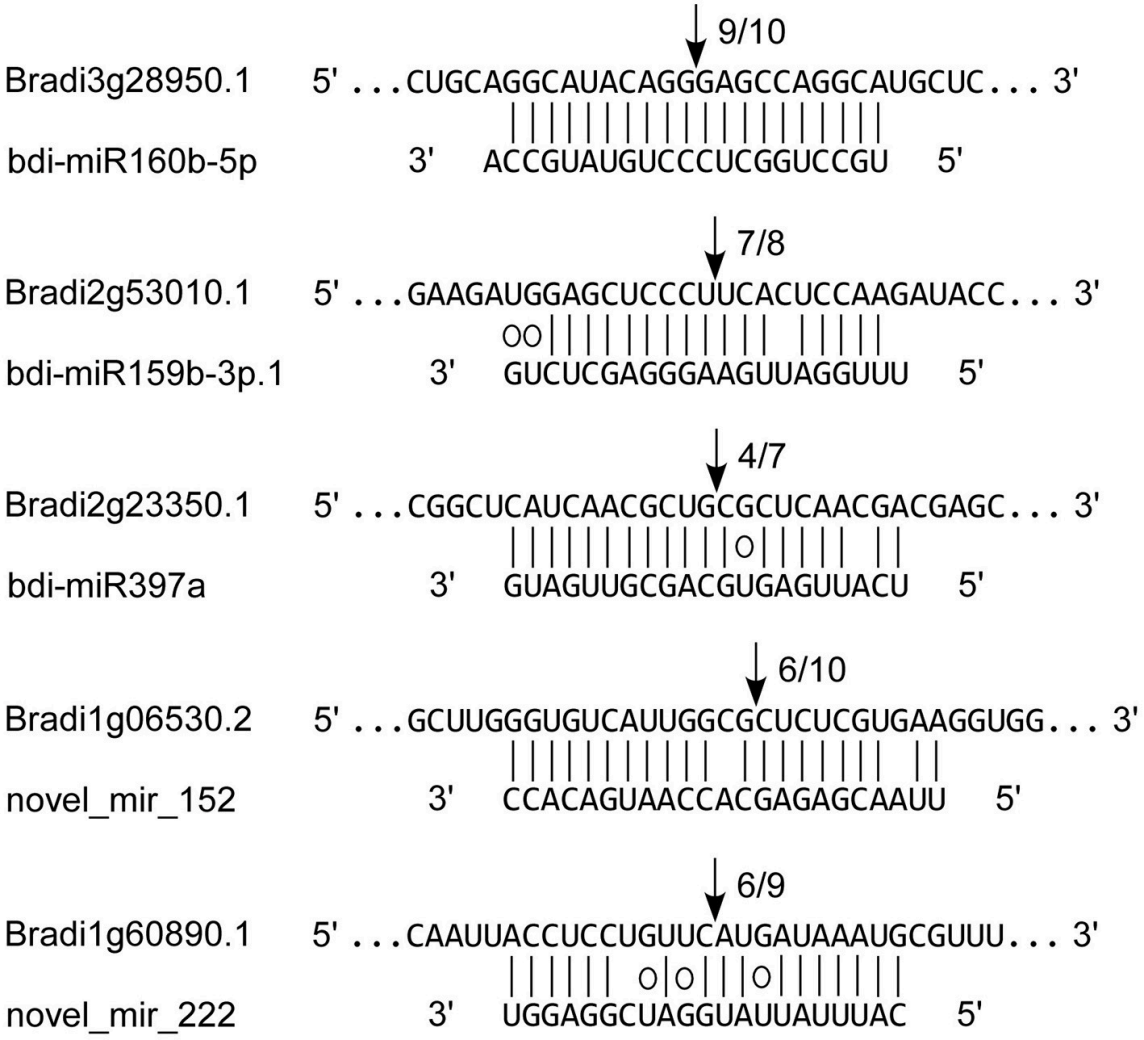
**Validation of known and novel miRNAs by RLM-5′-RACE**. Each top strand represents the miRNA complementary site on target mRNA, and each bottom strand represents the miRNA. Watson-Crick pairing (vertical dashes) and G:U wobble pairing (circles) are indicated. The arrows indicated the cleavage sites of target genes, and the numbers showed the frequency of cloned 5′ RACE products.

To verify the miRNA-guided target cleavage, RLM-5′-RACE experiment was performed to detect cleavage product of 3 known (bdi-miR160b-5p, bdi-miR159b-3p.1, and bdi-miR397a) and two novel bdi-miRNAs (novel_mir_152 and novel_mir_222). As shown in Figure 7, all five of the *B. distachyon* miRNAs guided the target cleavage, often at the tenth nucleotide (Figure 7).

## Discussion

To examine the H_2_O_2_-responsive miRNAs, two sRNA libraries were constructed from a mixture of 12-day old *B. distachyon* seedlings treated with 20 mM H_2_O_2_ and a control sample, and were then subjected to next-generation deep sequencing. We identified a set of known and novel H_2_O_2_-responsive miRNAs with notable expression pattern changes and we also provide a miRNA-mediated oxidative stress response PPI network. These results provide useful information for elucidating the response and defense mechanisms for H_2_O_2_ stress at the post-transcriptional level in plants.

Given that the expression of target genes is negatively regulated by miRNAs, the expression patterns of target genes always show an inverse correlation with those of miRNAs. However, our miRNA-target network showed that the regulatory mechanism of miRNA may be more complex. Some miRNAs can each regulate several target genes with the same function or different functions, and some genes can be regulated by several miRNAs. Among the known differential miRNAs in our study, some regulated two or more target genes with the same function, such as bdi-miR159a and bdi-miR169d/k, whereas more miRNAs, such as bdi-miR395, bdi-miR408, and bdi-miR528, regulated two or more target genes with different functions (Figure 4 and Table 1). Among the novel differential miRNAs, novel_mir_152 was downregulated (log2 fold change = −8.13) under H_2_O_2_ stress and mediated four target genes encoding a cell differentiation protein RCD1 homolog associated with RNA degradation and cell differentiation. The 20 potential genes regulated by novel_mir_120 are involved in various functions. Gene *Bradi2g55497*, which encodes transcription initiation factor TFIID subunit 12, was regulated by three novel miRNAs (novel_mir_98, novel_mir_120, and novel_mir_222). Two of them (novel_mir_98 and novel_mir_120) were downregulated under H_2_O_2_ stress, while novel_mir_222 was upregulated. Compared with novel_mir_98 and novel_mir_120, novel_mir_222 may have the opposite effect on the expression of *Bradi2g55497*. Furthermore, RT-qPCR analysis demonstrated that the final result of the antagonism is the downregulation of *Bradi2g55497* (Figure 4), so novel_mir_222 may play a main role under H_2_O_2_ stress. Four of the target genes of downregulated novel_mir_120 and upregulated novel_mir_222 are the same, which seems to imply that they are a pair of miRNAs with opposing effects. Additionally, novel_mir_91 and novel_mir_197 also exhibited the same pattern. Previous studies also found similar phenomenon. For example, genes *SPL* and *AP2*, encoded DNA-binding transcription factors, can be regulated by miR156 and miR172, which showed opposite expression patterns (Ding et al., 2014).

The upregulation of a miRNA always results in the aggravated degradation of its target gene in plants, and vice versa. These target genes of upregulated miRNAs may be associated with stress response, whereas the target genes of downregulated miRNAs are always involved in stress resistance. miR528, a monocot-specific miRNA, was upregulated under H_2_O_2_ stress (log2 fold change = 2.88). bdi-miR528 is also upregulated in Bd21 under drought stress (Bertolini et al., 2013) and in rice under H_2_O_2_ stress (Li et al., 2010). *Bradi3g19170*, the target gene of bdi-miR528, codes the E3 ubiquitin-protein ligase XBOS35, which may be involved in ubiquitination for proteasomal degradation and the ethylene (ET) signaling pathway during abiotic stress response (Sobeih et al., 2004; Prasad et al., 2010). Thus, miR528 may be a vital miRNA involved in water and oxidative stress response in monocots. As the largest MIR gene family in *B. distachyon*, the *MIR395* gene family includes 15 members. In our study, 13 members of the *MIR395* gene family were overexpressed under H_2_O_2_ stress and their target genes were *Bradi1g09030* (encoded ATP-sulfurylase 1), *Bradi1g24110* (encoded Ribosome-recycling factor), and *Bradi2g52840* (encoded disease resistance protein RGA4), which may be involved in oxidative stress response. Similar to our results, all of the identified *MIR395* gene family members are also upregulated under drought stress in *B. distachyon* (Bertolini et al., 2013). All of the target genes of bdi-miR397a, except *Bradi3g03407*, encode laccase, which plays an important role during the formation of lignin in the cell wall (Mayer and Staples, 2002); a previous study also confirmed that miR397 is a negative regulator of laccase genes (Lu et al., 2013). bdi-miR827-3p was upregulated under H_2_O_2_ stress, and a previous study (Jeong et al., 2013) found that this miRNA was also upregulated under phosphate starvation conditions in shoots. In contrast to our results, a previous study showed that miR397 and miR827 were downregulated in rice by H_2_O_2_ treatment, which indicates that the miRNA response to the same abiotic stress occurs in a genotype/species-dependent manner (Zhang, 2015).

In plants, miRNAs participate in the response to abiotic stress by mediating key components of complex gene networks (Zhang, 2015). Many of the target genes of miRNAs identified in our study are TFs, which is consistent with previous studies (Jones-Rhoades et al., 2006; Baev et al., 2011; Zhang, 2015). Thus, a miRNA can indirectly regulate the expression of downstream target genes through regulation of the expression of TFs. Previous studies revealed that the mechanism of the MYB TF involved in gibberellic acid (GA) signal transduction was regulated by miR159 (Achard et al., 2004; Alonso-Peral et al., 2010). In our study, bdi-miR159a/b (target genes *Bradi2g53010* and *Bradi1g36540*, two *MYB genes*) was upregulated under H_2_O_2_ stress, which is consistent with the results in rice and *B. distachyon* under drought stress (Zhou et al., 2010; Bertolini et al., 2013). Auxin response factors (ARFs) are a class of auxin-responsive TFs mediated and regulated by miR160 (Mallory et al., 2005). miR160 regulates the expression of *ARF* genes by combining with the complementary sites of a non-coding region of *ARF* genes (Rhoades et al., 2002). In our study, gene *ARF22* was targeted by four downregulated members of the bdi-MIR160 family (Table 1). Interestingly, gene *ARF5* was targeted by the upregulated bdi-miR397a, instead of bdi-miR160. In addition, the expression of some miRNAs can also be regulated by TFs through specific binding to the promoter region of the miRNA. For example, miR160 can be transcriptionally regulated by proteins ARF6 and ARF17 in *Arabidopsis* (Gutierrez et al., 2009). Thus, there are fine tuning mechanisms in plants to modulate gene expression through miRNA-TF-mediated feedback loops (Meng et al., 2011; Iyer et al., 2012). bdi-miR169 was significantly downregulated under H_2_O_2_ stress in our study and a previous study in rice (Li et al., 2010); its target gene encodes a nuclear TF Y subunit A-2 (NF-YA-2) or NF-YA-4, which belongs to the CCAAT binding TF family. In *Arabidopsis*, the upregulation of *NF-YA* gene depends on the regulation of miR169 under drought stress (Li et al., 2008). Thön et al. (2010) found that CCAAT-binding TFs are involved in the response to oxidative stress. Therefore, miR169 may play a crucial role in the process of resistance to oxidative stress in plants. The network analysis showed that NF-YA could interact with V-type proton ATPase subunit d (encoded by *Bradi2g42100*), transcription initiation factor TFIID subunit 12 (encoded by *Bradi2g55497*), 2-oxoglutarate-dependent dioxygenase DAO (encoded by *Bradi2g24715*), and TF MYB (encoded by *Bradi2g53010* and *Bradi1g36540*). One of the target genes of the upregulated bdi-miR408-5p encodes TF TCP15, whose activity is inhibited by oxidative stress (Viola et al., 2013). Overexpression of miR408 in *Arabidopsis* can improve its tolerance to oxidative stress (Ma et al., 2015).

Among the differentially expressed novel miRNAs, novel_mir_160, a potential new member of the *bdiMIR171* gene family, was downregulated (log2 fold change = −2.12) under H_2_O_2_ stress. miR171 is also significantly downregulated under drought stress in rice and potato (Zhou et al., 2010; Hwang et al., 2011). Although we did not identify the target genes of this potential novel *bdiMIR171* gene family member, a previous study in potato showed that GRAS family TF, involved in development and stress responses, such as drought stress (Ma et al., 2010), is the putative target gene of miR171 (Hwang et al., 2011). The pre-miRNA of novel_mir_162 was the same as the precursor of bdi-miR395c, which may be a novel member of the *bdiMIR395* family. Similar to other known bdi-miR395 family members, this miRNA was also upregulated (Table 2). In addition, novel_mir_40 was identified as a novel member of the *bdiMIR169* family, which was also downregulated (log2 fold change = −10.28), as were the five known differentially expressed *bdiMIR169* family members (Table 1). In contrast to typical members of the *bdiMIR169* family, whose target genes always encode NF-YA TFs, the target gene of novel_mir_40 is *Bradi1g25517*, which encodes glucan endo-1,3-beta-glucosidase 3.

H_2_O_2_ can trigger various phytohormone signaling pathways involved in abiotic and biotic stress responses and there are complex crosstalks among different phytohormone signaling pathways (Harrison, 2012; Saxena et al., 2016). Recent study found out some immune hormone marker genes involved in salicylic acid (SA), jasmonic acid (JA) and ET signaling pathways in *B. distachyon* (Kouzai et al., 2016). In our study, we also found some genes related with phytohormone signaling pathways were the target of miRNAs. For example, the two *MYB* genes involved in GA pathway were regulated by bdi-miR159a/b, *ARF22*, and *ARF5* genes involved in auxin pathway were the target of bdi-miR160 and bdi-miR397a, respectively, and the *E3 ubiquitin-protein ligase XBOS35* and *ethylene-responsive transcription factor RAP2-3* genes related with ET signaling pathways were mediated by bdi-miR528 and novel_mir_120/novel_mir_222, respectively.

## Conclusion

In this study, we sequenced and analyzed the sRNA of model plant Bd21 seedlings under H_2_O_2_ stress and normal growth conditions using large-scale sequencing technology. Finally, we identified 39 known and 221 novel miRNAs, of which 31 known miRNAs and 30 novel miRNAs were involved in H_2_O_2_-stress response and resistance. Moreover, RT-qPCR analysis of several representative miRNAs and their target genes and cleavage site analysis through 5′ RACE validated our sequencing and bioinformatic results. The PPI network mediated by miRNAs revealed the regulation mechanism of signal transduction and oxidative stress resistance under H_2_O_2_ stress. Further analysis of the differentially expressed miRNAs and their target genes will help us understand the mechanism of oxidative stress response and tolerance in plants.

## Author contributions

DL carried out all experiments, data analysis and wrote the manuscript. SZ contributed the RLM-5′-RACE experiment. GZ, YB, and GC performed the RNA extraction and RT-qPCR, CH and ZY conducted GO annotation and enrichment analyses. DL and YY conceived the study, participated in the design and coordination, and in interpretation of the dataset. All authors read and approved the final manuscript.

### Conflict of interest statement

The authors declare that the research was conducted in the absence of any commercial or financial relationships that could be construed as a potential conflict of interest.

## Supplementary material

Figure S1 **First base bias analysis of the novel miRNAs. (A)** First base bias of all novel Bd-miRNAs in the two libraries (CS and TS); **(B)** First base bias of 20-, 21-, 22-, and 23-nt novel Bd-miRNAs in the two libraries (CS and TS).

Figure S2 **GO annotation of all potential miRNA target genes**. Biological process, molecular function, and cellular component categories were displayed.

Table S1 **All the RT-qPCR and RACE primers used in this study**.

Table S2 **The distribution of sRNAs in each class**.

Table S3 **The known *B. distachyon* miRNAs identified in this study**.

Table S4 **The novel *B. distachyon* miRNAs identified in this study**.

Table S5 **List of known and novel miRNAs and their target genes. (A)** List of known miRNAs and their target genes; **(B)** List of novel miRNAs and their target genes.

## Abbreviations

miRNA: microRNA
RT-qPCR: Reverse transcription-quantitative polymerase chain reaction
ROS: Reactive oxygen species
H_2_O_2_: Hydrogen peroxide
SOD: Superoxide dismutase
TF: Transcription factor
RT-qPCR: Reverse transcription quantitative polymerase chain reaction
sRNA: Small-RNA
MFE: Minimum of free energy
GO: Gene Ontology
FDR: False discovery rate
PPI: Protein-protein interaction
CS: Control seedlings
TS: H_2_O_2_-treated seedlings
ARF: Auxin response factor
ET: Ethylene
SA: Salicylic acid
JA: Jasmonic acid.
