# Supplementary figures and images for: High-Throughput Sequencing Reveals H2O2 Stress-Associated MicroRNAs and a Potential Regulatory Network in Brachypodium distachyon Seedlings
Source: Front Plant Sci. 2016 Oct 20;7:1567. doi: 10.3389/fpls.2016.01567 (PMC5071335; doi:10.3389/fpls.2016.01567)

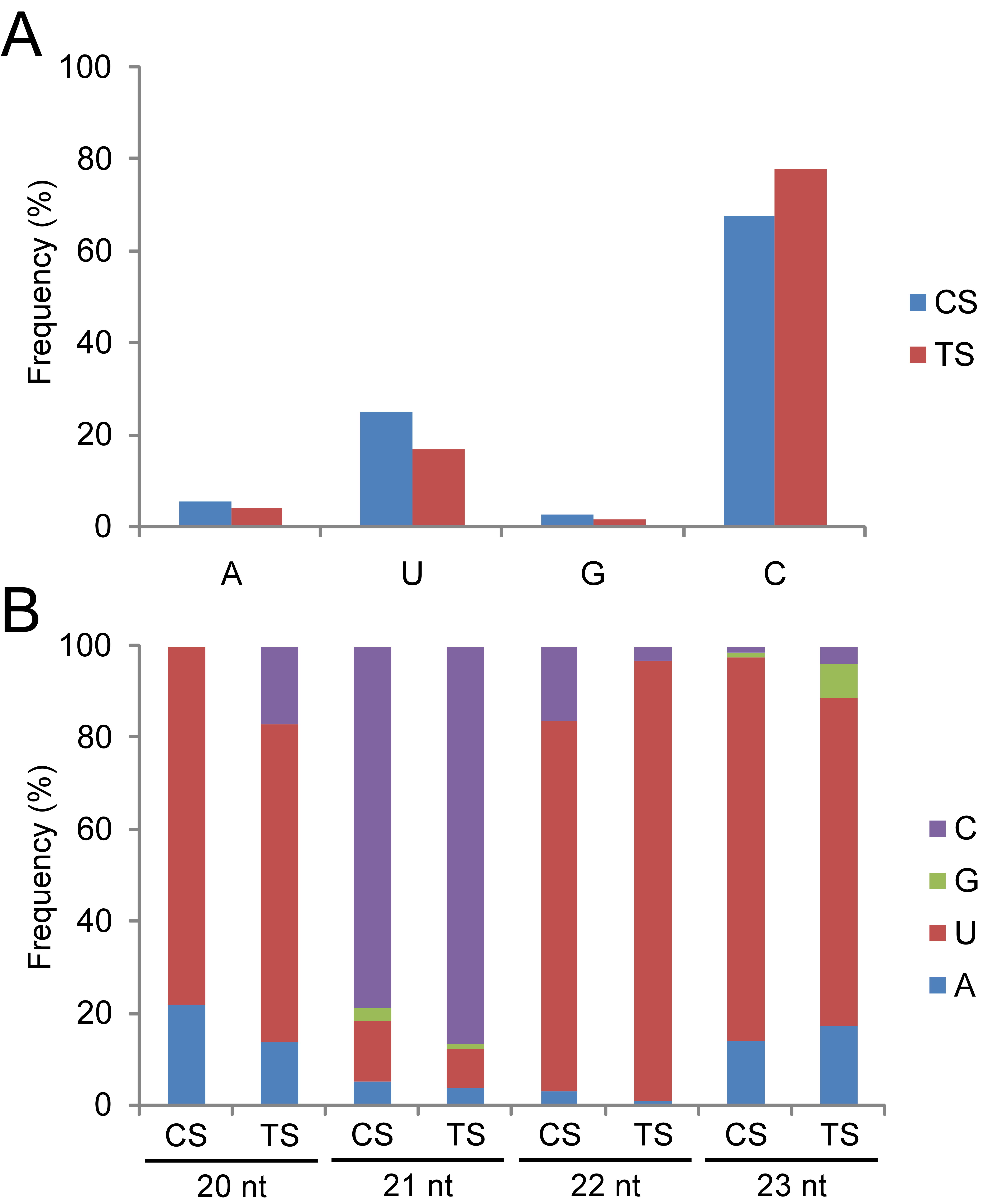

Supplement: Figure S1 — First base bias analysis of the novel miRNAs. (A) First base bias of all novel Bd-miRNAs in the two libraries (CS and TS); (B) First base bias of 20-, 21-, 22-, and 23-nt novel Bd-miRNAs in the two libraries (CS and TS). [file Image1.JPEG]

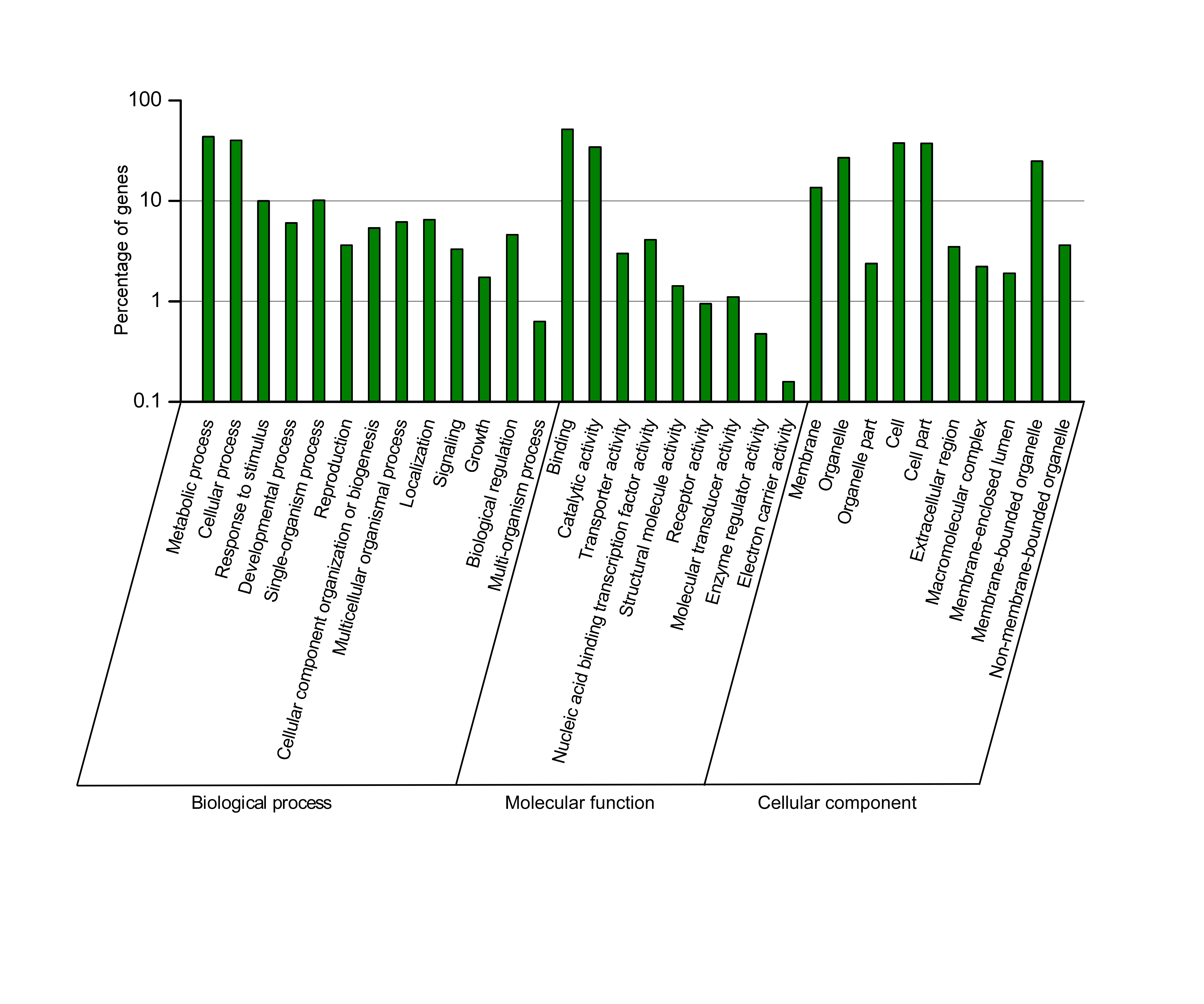

Supplement: Figure S2 — GO annotation of all potential miRNA target genes. Biological process, molecular function, and cellular component categories were displayed. [file Image2.JPEG]
