# Supplementary material for: High-Throughput Sequencing Reveals H2O2 Stress-Associated MicroRNAs and a Potential Regulatory Network in Brachypodium distachyon Seedlings
Source: Front Plant Sci. 2016 Oct 20;7:1567. doi: 10.3389/fpls.2016.01567 (PMC5071335; doi:10.3389/fpls.2016.01567)
Supplement: Table S2 — The distribution of sRNAs in each class. [file Table2.DOCX]

**Table S2. The distribution of sRNA in each class**

| **Class** | **CS** | | **TS** | | **CS** | | **TS** | |
| --- | --- | --- | --- | --- | --- | --- | --- | --- |
|  | **Unique sRNA** | **%** | **Unique sRNA** | **%** | **sRNA Count** | **%** | **sRNA Count** | **%** |
|  |  |  |  |  |  |  |  |  |
| Total | 3830474 | 100% | 3548088 | 100% | 17811109 | 100% | 17708762 | 100% |
| exon_antisense | 16102 | 0.42% | 14312 | 0.40% | 24126 | 0.14% | 20770 | 0.12% |
| exon_sense | 142000 | 3.71% | 122298 | 3.45% | 198342 | 1.11% | 169792 | 0.96% |
| intron_antisense | 51223 | 1.34% | 43827 | 1.24% | 124955 | 0.70% | 104046 | 0.59% |
| intron_sense | 83821 | 2.19% | 72775 | 2.05% | 209913 | 1.18% | 170688 | 0.96% |
| miRNA | 1263 | 0.03% | 1309 | 0.04% | 1101886 | 6.19% | 1496740 | 8.45% |
| rRNA | 469843 | 12.27% | 466257 | 13.14% | 6745497 | 37.87% | 6905172 | 38.99% |
| repeat | 203405 | 5.31% | 177054 | 4.99% | 336422 | 1.89% | 276823 | 1.56% |
| snRNA | 4433 | 0.12% | 4949 | 0.14% | 18249 | 0.10% | 26231 | 0.15% |
| snoRNA | 1225 | 0.03% | 1471 | 0.04% | 3084 | 0.02% | 4140 | 0.02% |
| tRNA | 83663 | 2.18% | 98761 | 2.78% | 1575045 | 8.84% | 1469726 | 8.30% |
| unann | 2773496 | 72.41% | 2545075 | 71.73% | 7473590 | 41.96% | 7064634 | 39.89% |
